# Acute Fulminant Group A Beta-Hemolytic Streptococcus-Associated Carditis: A Case Report and Literature Review

**DOI:** 10.7759/cureus.27282

**Published:** 2022-07-26

**Authors:** James Allen, Christine Munoz, Alla Byakova, Roman Pachulski

**Affiliations:** 1 Internal Medicine, St. John's Episcopal Hospital, New York, USA; 2 Cardiology, St. John's Episcopal Hospital, New York, USA

**Keywords:** antistreptolysin o, troponin, post-streptococcal carditis, acute rheumatic fever, group a β hemolytic streptococci

## Abstract

Group A beta-hemolytic streptococcus (GAS) is a gram-positive bacteria found in the upper respiratory tract that can cause disease with a wide gamut of symptoms ranging from pharyngitis to peritonsillar abscess, pneumonia, meningitis, and acute rheumatic fever (ARF). The primary goal of antibiotic therapy is to prevent complications of the primary infection such as ARF. ARF is defined by the revised Jones criteria. The Jones criteria have been modified to account for the moderate- to high-risk populations. The mechanism of the development of ARF from pharyngitis is not well understood, but the leading theory is molecular mimicry. The host’s own immune system that responds to bacterial virulence factors develops autoantibodies that attack the host tissue. ARF typically develops two to four weeks post pharyngitis. Markers such as antistreptolysin O rise by week 2-3. The rapid streptococcal antigen is often negative by the time ARF develops.

We present a case of a 23-year-old male with no past medical history who presented with a chief complaint of fever and sore throat for one week associated with new-onset chest pain. The patient had a fever with normal blood pressure. Labs showed mild leukocytosis, elevated troponin I, and positive Group A strep polymerase chain reaction (PCR). He was initially treated with aspirin 81 mg, antibiotics, and non-steroidal anti-inflammatory drugs (NSAIDs) in the emergency room. The patient was subsequently started on prednisone 60 mg as he showed no clinical improvement. His initial echocardiography (ECHO) showed a left ventricular ejection fraction (LVEF) of 55%. Repeat ECHO showed LVEF of 45% with regional wall motion abnormalities (RWMA). His cardiac troponin continued to rise with EKG changes on day 7. With the addition of steroids, the patient’s clinical symptoms, as well as EKG and ECHO findings, improved. The patient was discharged with penicillin benzathine for 12 weeks.

Case reports of acute carditis presenting concomitantly with pharyngitis are limited. The diagnosis of post-streptococcus complications relies on antistreptolysin O titer (ASOT) serology. With the increased availability of more acute diagnostic markers such as PCR, troponin, and ECHO, GAS confirmation can potentially be obtained within one hour and maybe in the future in the diagnosis of early-onset ARF.

## Introduction

Group A beta-hemolytic streptococcus (GAS) is a ubiquitous gram-positive bacteria that may be found as a colonizer of the upper respiratory tract and yet manifest disease in a variety of ways in the human body, ranging from mild to severe disease. GAS is the most common cause of acute bacterial pharyngitis. However, it has also been known to cause many suppurative complications including sinusitis, otitis media, impetigo, peritonsillar abscess, tonsillopharyngeal cellulitis, osteomyelitis, pneumonia, and bacteremia as well as more potentially life-threatening infections such as meningitis, necrotizing fasciitis, and myonecrosis. GAS infections are also associated with non-suppurative complications including post-streptococcal reactive arthritis, scarlet fever, acute glomerulonephritis, acute rheumatic fever (ARF), and streptococcal toxic shock syndrome (TSS). Although GAS pharyngitis is often self-limiting, the primary goal of antibiotic therapy is to prevent these complications while limiting the duration and spread of the primary infection [1,2].

ARF has various manifestations described as major or minor by the revised Jones criteria. The five major manifestations of ARF are (1) carditis, clinical or subclinical; (2) migratory polyarthritis of the large joints; (3) central nervous system involvement (Sydenham chorea); (4) subcutaneous nodules, and (5) erythema marginatum. Minor criteria include polyarthralgia, fever, elevated acute phase reactants (erythrocyte sedimentation rate [ESR] and C-reactive protein [CRP]), and prolonged PR interval on ECG. Although minor manifestations are often present, they are non-specific. In a person with proven preceding GAS infection (discussed below), two major or one major with two minor criteria are sufficient for the diagnosis of ARF. Note that cardiac or joint involvement may only be counted as either a major or minor criterion, not both. Individuals with a previous history of ARF are at risk of recurrent episodes with a greater likelihood of developing cardiac involvement; Jones criteria are modified for this group such that the presence of three minor criteria would also be sufficient for diagnosis. The revised Jones criteria have also been modified for patients from moderate- to high-risk populations to account for the risk of underdiagnosis in populations with increased prevalence. Jones criteria have been relaxed in this group to accept monoarthritis as a major criterion and monoarthralgia, low-grade fever, and lower cutoff values for acute phase reactants as minor criteria [2-4].

GAS boasts a host of varied and evolving virulence mechanisms involved in the evasion of host immune responses, stimulation of host cell destruction, tissue degradation, adhesion, and invasion into tissues. See Appendix A for more detailed GAS virulence factor information [5]. The mechanism by which GAS pharyngitis precipitates ARF is not well understood but thought to occur via the host’s own immune response to bacterial virulence factors. Antibodies made to protect the host against the pathogen instead act as autoantibodies causing damage to host tissues or marking cells for destruction; myosin, laminin, and keratin have been identified as potential autoantibody targets via molecular mimicry [6,7]. Antibodies against M protein, a structural GAS protein thought to protect the bacteria from phagocytosis, have been extensively researched in the development of carditis in ARF by cross-reactivity with myosin [4,5]. ARF usually occurs in the two to four weeks following an episode of GAS pharyngitis detected by the classic clinical and possibly outdated “Jones” criteria and antibody production beginning at one to three weeks and peaking at three to five weeks post-primary infection. The latency period between the primary infection and ARF further bolsters the idea that it is an antibody-mediated reaction. A single severe episode of ARF or repeated bouts of inflammation with fibrinous repair causes the valvular disease seen in rheumatic heart disease (RHD), the most common cause of acquired valvular disease in the world [3,4].

Other virulence factors, such as streptolysin O and deoxyribonuclease B, warrant further discussion. In addition to their roles in bacterial virulence during GAS pharyngitis and stimulation of aberrant host immune response responsible for the pathogenesis of ARF, the antistreptococcal antibodies formed against them have become clinically useful diagnostic tools. Antistreptolysin O (ASO) titer levels rise and peak in the timeframe coinciding with ARF presentation, providing measurable evidence of recent GAS infection. Though acetylsalicylic acid (ASA) is potentially helpful, the marker’s usefulness is limited by the delay of onset by two to three weeks. Throat cultures have a high false-negative rate of 40%, even acutely during that period due to resolving bacterial infection. Rapid streptococcal antigen tests are also often negative, requiring further testing in scenarios of high suspicion. Anti-deoxyribonuclease B (ADB) titers can be used in a similar fashion, with peak levels occurring at six to eight weeks post-GAS pharyngitis. It is becoming a common practice to collect ASO and/or ADB titers at first clinical suspicion of ARF and then two weeks later for comparison [2,3,5,8]. Evidently, no diagnostic marker was previously able to reliably detect GAS during an acute infection, further demonstrating the benefits of modern, acute markers such as PCR and troponins.

## Case presentation

A 23-year-old male with no significant past medical history presented complaining of fever and sore throat for the past week with new onset of chest pain, which prompted the emergency room visit. Chest pain was described as non-radiating, retrosternal pressure sensation, 6/10 in severity, exacerbated upon movement, deep inspiration, and exertion. Associated symptoms included generalized weakness, occasional myalgia, and polyarthralgia. The patient denied diaphoresis, palpitations, rashes, nausea, vomiting, dysuria, hematuria, chills, sick contacts, recent travel, or experiencing similar symptoms in the past. The patient also denied seeking medical treatment in the past week, taking medications, smoking, alcohol, or using illicit substances. Family history was non-contributory, with no reported history of atherosclerotic heart disease.

Upon arrival, the patient was awake, alert, and oriented x3 and appeared in no acute distress. The patient was febrile (101.3°F) and hemodynamically stable; the remainder of the vital signs were unremarkable. Upon physical examination, the patient was warm to touch, had moist mucous membranes, non-injected sclera, and no erythema or exudate of the posterior oral pharynx. The cardiac exam showed a normal S1/S2, 2/6 mid-diastolic murmur, no S3, audible S4, and strong pulses equal in upper and lower extremities bilaterally. The lungs were clear to auscultation bilaterally with no adventitious breath sounds. The patient's abdomen was soft, non-tender, and with normal bowel sounds. No lymphadenopathy, rashes, extensor nodules, or involuntary movement were noted.

**Figure 1:**
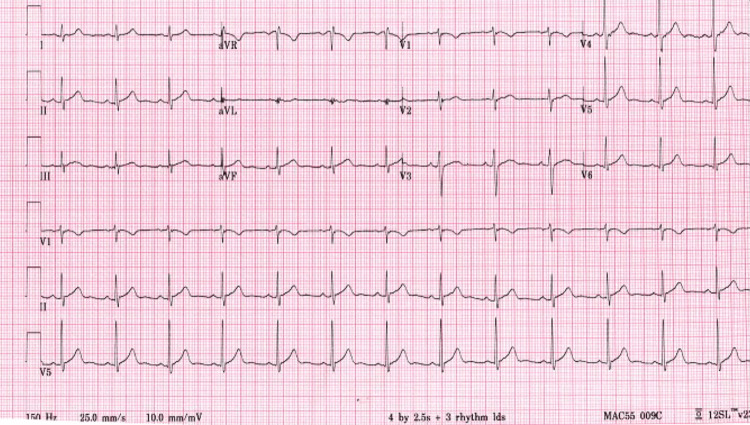
Day 1 electrocardiogram showing normal sinus rhythm

Initial laboratory studies revealed leukocytosis (12.2 K) with neutrophilic predominance (75.7%), elevated absolute neutrophil count (9.19), unremarkable comprehensive metabolic panel, elevated cardiac troponin I (7.33), elevated creatine kinase-MB (CK-MB%) (5.2), and ESR (11). Strep Group A PCR-positive, EKG revealed normal sinus rhythm (80 bpm) with normal PR interval and no acute ST-T shift. The chest x-ray was unremarkable (Figure 1). The following serologies were negative: Coxsackie, echovirus, mumps, gonorrhea, parvovirus, mono-screen, HIV-1 and 2, toxoplasma, influenza A and B, and Lyme. Collagen vascular markers were also negative as follows: rheumatoid factor (RF)-19, antinuclear antibody (ANA) 1:80, proteinase 3, antiproteinase, myeloperoxidase, Smith-antibody, ribonucleoprotein (RNP) antibody, double-stranded DNA (ds DNA) antibody, and chromatin antibody. Urine was negative for protein and toxins, including opiates, methadone, barbiturates, phencyclidine, amphetamine, and benzodiazepines. Serum electrolytes and renal and hepatic function were unremarkable. Blood cultures were negative. The patient received aspirin 81 mg and antibiotics (Abx) (ceftriaxone 2 g plus vancomycin 1 g) while in the emergency department. ASA was increased to 325 q6hr (every 6 hours) the following day. Two doses of benzathine penicillin 1.2 million units intramuscular (IM) were administered on days 2 and 3 and continued weekly for four weeks. As there was no clinical improvement, prednisone 60 mg was started on day 6 with a 12-day taper.

**Figure 2:**
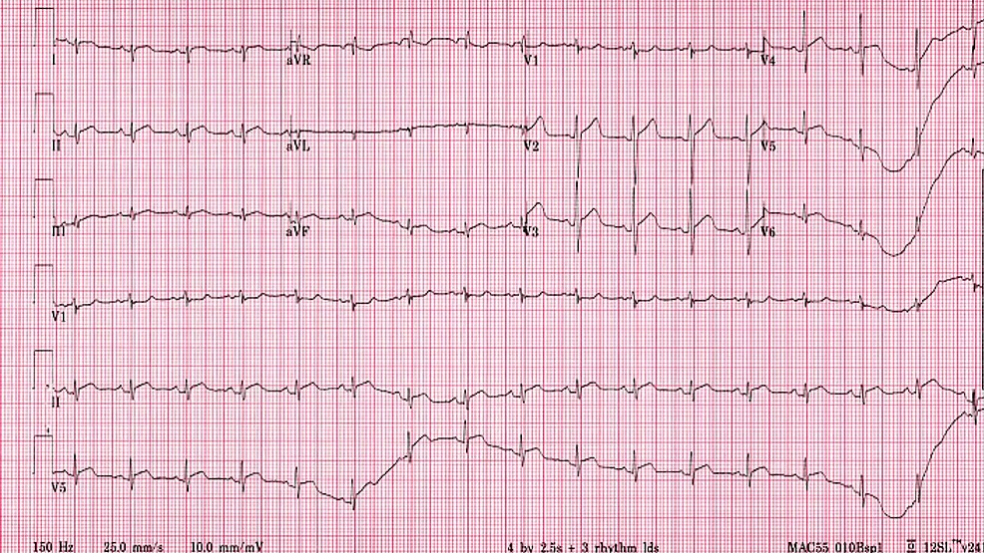
Day 6 electrocardiogram showing diffuse ST elevations

**Figure 3:**
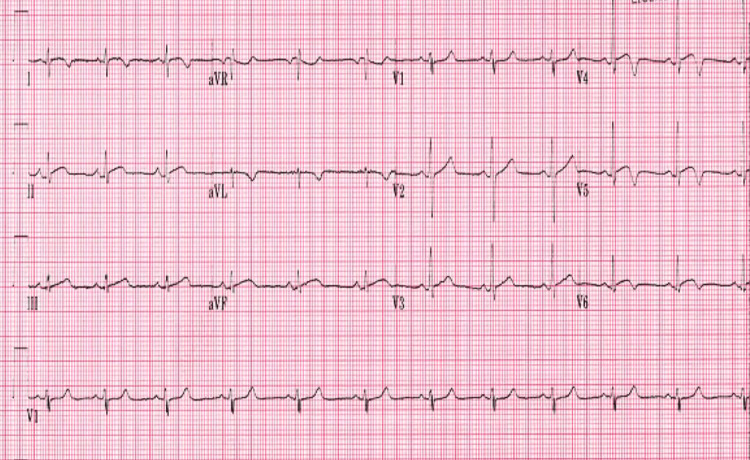
Day 7 electrocardiogram showing diffuse ST elevation with lateral wall T-wave inversions

**Figure 4:**
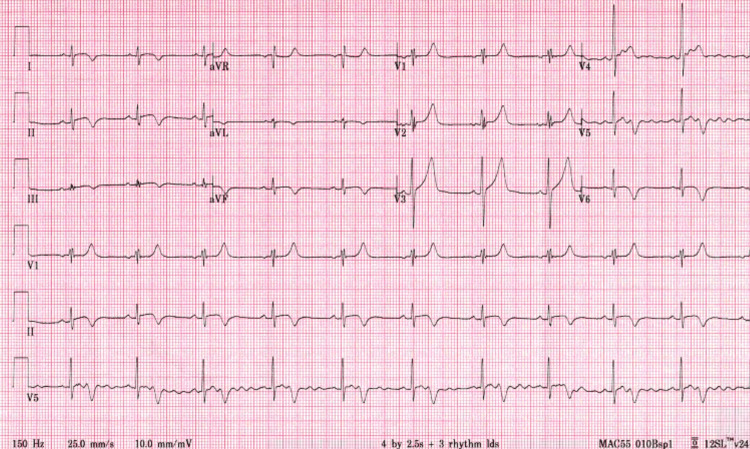
Day 28 electrocardiogram showing global T-wave inversions

Throughout the hospital course, there was an evolution of symptomology, physical exam findings, serologic inflammatory markers, EKG findings, and transthoracic ECHO (TTE) findings. Retrosternal chest pain was present on admission and persisted intermittently for the next three days. The patient's fever peaked on day 5 (102.4°F) and was afebrile by day 7. Diastolic murmur was only present on initial presentation, and pericardial rub, initially absent, presented on day 6 and persisted until day 9. Cardiac troponins continued to rise and peaked (10.0) on day 3 and were normal by day 7. CRP was elevated (5.1) on day 2, and ESR peaked (18.2) by day 5. Diffuse ST elevation was noted on days 3-6 (Figure 2), and diffuse ST elevation along with new lateral wall T-wave inversions was noted on days 7-8 (Figure 3), with a return to baseline on day 9. On the follow-up visit, day 28, EKG revealed global T-wave inversions (Figure 4).

**Figure 5:**
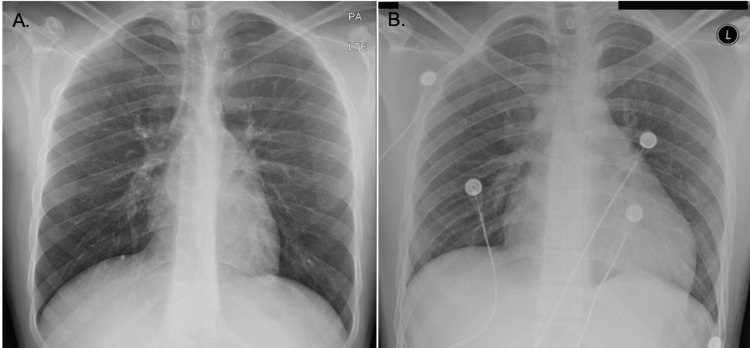
(A) Chest x-ray on the day of presentation, normal. (B) Chest x-ray on day 5 of admission showing enlargement of the cardiac silhouette.

These EKG findings may mimic myocardial infarction (MI). Key differentiations are that the hallmark EKG changes for pericarditis are global ST-T changes, PR depression, and sequential ST elevation followed by T-wave inversion (vs. MI where ST and T changes occur simultaneously). TTE on day 1 revealed LVEF of 55% with anterior mitral valve leaflet thickening and trivial posterior pericardial effusion. Repeat TTE on day 6 revealed decreased LVEF to 45% with regional wall motion abnormalities (RWMA). On day 28, pre-discharge TTE revealed a return to normal LVEF of 65% and resolution of pericardial effusion. See Figure 5 and Table 1 for a detailed summary of the clinical course.

**Table 1:** Evolution of clinical measures RSCP: Retrosternal chest pain; BP: Blood pressure; HR: Heart rate; ESR: Erythrocyte sedimentation rate; CRP: C-reactive protein; GAS-PCR: Group A beta-hemolytic streptococcus-polymerase chain reaction; ASA: Acetylsalicylic acid; TWI: T-wave inversion; ALMV: Anterior leaflet of the mitral; RWMA: Regional wall motion abnormalities; ST Elev: ­ST elevation; ECHO: Echocardiography; EF: Ejection fraction.

Day	1	2	3	4	5	6	7	8	9	28
RSCP	Present	Present	Present	Present	Absent	Absent	Absent	Absent	Absent	Absent
BP	114/62	98/54	106/63	103/51	97/51	101/62	113/64	112/67	124/59	136/84
HR	83	101	100	111	120	86	60	71	55	77
Temp (°F)	101.3	102	100.5	101.4	102.4	102.1	96.9	96.8	96.4	97.6
Diastolic murmur	Present	Absent	Absent	Absent	Absent	Absent	Absent	Absent	Absent	Absent
Pericardial rub	Absent	Absent	Absent	Absent	Absent	Present	Present	Present	Present	Present
WBC (10)	12.2	10.7	13.6	16.4	17.2	21.4	19.2	16.6	14.3	6.8
Neut% (74)	76	76	77	84	75	81	87	78	75	55
Lymph% (19)	16	15	12	10	16	11	9	17	19	30
Trop (0.036)	7.33	7.31	10	8.73	7.19	5.85	0.81			<0.012
ESR (15)		11			77		77	70	49	4
CRP (0.9)	5.1				18.2					<0.5
Ca (8.4)	9.7	8.9	9.6	8.4	8.8	8.7		9.2	9.4	10.2
Alb (3.5)	4.3	3.5	3.5	3.1	2.8	2.8		2.8	3.3	
GAS PCR	+									
EKG ST-T	ST	ST	­ST Elev			­­­­­­­ST Elev­	ST Elev w/ lat TWI­­	ST Elev w/ lat TWI­­	ST	Global TWI
ECHO EF	55					45		65		65
ECHO Eff	Small					­Increased­				Effusion
ECHO other	ALMV					RWMA				
ASA	81	1300	1300	1300	1300	1300	1300	1300	1300	325
Prednisone						60	55	50	45	0

The patient's initial presentation was concerning for ARF, fulfilling one major criterion (carditis-elevated troponins with early/subclinical mitral valve disease on TTE) and two minor criteria (elevated CRP > 3.0 and fever of 101.3°F on admission). Evidence for pre-existing GAS was present. Upon admission, the patient initially received ceftriaxone 2 g and vancomycin, then changed to benzathine penicillin 1.2 million units IM. The patient received high-dose aspirin (81 mg in ED, followed by 1300 mg the next day) daily. Despite high-dose NSAIDs and dose-appropriate antibiotics, the patient showed minimal improvement in fever, heart rate, and troponin, with a new audible monophonic rub, enlarged pericardial effusion, new regional inferior wall motion abnormality, and reduction in global LVEF to 45% on echo. The patient was started on prednisone (60 mg, steadily tapered by 5 mg daily) on day 6. With this addition, the patient's symptoms improved, fever subsided, tachycardia resolved, and troponin level decreased to normal within 24 hours. Upon discharge, the patient was continued on prophylactic benzathine penicillin for 12 weeks monthly to prevent/reduce the risk for future endocarditis as an outpatient.

## Discussion

Distinguishing features of this case include (1) initial presentation of fever, sore throat, and chest pain absent arthritis; (2) shortened latency period from pharyngitis to pancarditis; (3) NSAID-refractory fever, leukocytosis, inflammatory markers, and troponin; and lastly (4) glucocorticoid-responsive fever, inflammatory markers, troponin, and systolic dysfunction.

There are only a handful of similar case reports of acute carditis in the setting of concomitant GAS pharyngitis worldwide. All but two of these cases were male, further suggesting that the pathogenesis of ARF is autoimmune in nature. Nearly, all of the cases reviewed reported shortened latency periods of three to seven days between the onset of sore throat and chest pain. Additionally, many did not present with arthritis, as in this case. Due to this deviation from the classically described ARF and inability to meet the Jones criteria (on presentation), many have termed this presentation as non-rheumatic streptococcal carditis or non-rheumatic streptococcal pharyngitis-associated myocarditis (SPAM), theorizing non-immune-mediated pathophysiological mechanisms [9-13]. Within the clinicopathological classification of myocarditis developed by Lieberman et al. (using the Dallas criteria), our case is most similar to the fulminant subtype in overall clinical course and recovery; however, the LV dysfunction and hemodynamic compromise were less severe [14]. We postulate that evaluation of the patient earlier in the disease process and/or inability to recognize the need for the use of the modified Jones criteria (recommended for moderate-/high-risk populations) has led to underdiagnosis of this phenomenon of acute fulminant GAS-associated carditis. With more rapid methods of detection, such as PCR and troponin (highly sensitive to myocardial damage), earlier detection can be made.

Chaudhuri et al., in the literature review of papers mentioning “non-rheumatic” streptococcal myocarditis, noted that this presentation appears to occur mostly in young males on beta-lactam antibiotic treatment [9]. They postulated that this might precipitate myocarditis in rare instances via mass toxin and bacterial cell wall fragments released during cell lysis (though there is little evidence to support this theory). An alternative hypothesis is high toxin release in the setting of high bacterial load in the stationary phase rendering antibiotics less effective [9]. However, all patients in these cases, and other similar cases we reviewed, were treated with antibiotics (penicillin class) and showed improvement instead of worsening course as would be expected with these proposed pathophysiological mechanisms. Additionally, the majority of cases reviewed reported positive ASO titers, albeit some weakly positive as in this case, suggesting a similar disease mechanism to ARF [9-13]. While other groups reported the presence of antibodies with ASO titers < 200 U/mL and positive rapid strep on presentation, repeat ASO titers were not reported [15,16]. Popescu et al. similarly reported ASO titer < 200 U/mL on admission with a three-fold increase on repeat titers by the time of discharge (approximately six days) [17]. It may be that the shortened latency period from pharyngitis to carditis seen in our patient and reported in similar cases results from a repeat (memory) immune response with patients previously exposed to GAS. During these repeated antigen presentations, the host immune response is more rapid, with shortened latency and a more pronounced overall response.

Previously, the diagnosis of post-streptococcal-related sequela, including carditis, relied on inaccurate and delayed tests such as throat culture and ASOT serology for GAS confirmation. With the advent and availability of more sensitive metrics, such as PCR with flocked swab sampling, GAS confirmation can now be obtained within one hour with 96% sensitivity and 98% specificity [18,19]. While ECG is beneficial and cost-effective in monitoring patients for cardiac involvement in ARF, the use of early ECGs in patients with GAS pharyngitis, even in the absence of cardiac symptoms, has been recognized. The use of ECG has been used in the description of cases showing similar disease phenomena by many groups, including what may possibly be the first characterization of this presentation in 1947 [17,20,21]. With the continued improvements in tests, decreased costs, and increased portability, as with troponin and echocardiography, their application in evaluating patients presenting with GAS pharyngitis reporting chest pain should be considered.

Timely recognition of this acute fulminant presentation of GAS-associated carditis allows for prompt treatment and reduction of the inflammatory/destructive process. While historical studies have previously suggested no benefit of antibiotic treatment, likely a consequence of delayed detection, more recent studies have shown penicillin treatment to reduce post-streptococcal pancarditis (when initiated within nine days of pharyngitis onset) and the efficacy of prophylactic treatment in the prevention of recurrent GAS infection and late cardiac sequela [22,23]. Similarly, new studies have proven the efficacy of glucocorticoid (GC) treatment in GAS-associated carditis (in ARF) with the use of GAS PCR, troponin, and ECHO [24-29]. Older studies that suggested GCs are ineffective used the presence of late audible murmur as the endpoint measure of permanent valvular disease [30-32]. In our case, the course of what we term acute fulminant GAS-associated carditis was dramatically foreshortened and resolved with glucocorticoids; within 24 hours of initiation of GCs, there was ST-T resolution and troponin collapse, with restored LVEF and resolution of RWMA within 48 hours of GC commencement.

In their literature review, Popescu et al. showed similar clinical improvement with the initiation of IV penicillin and dexamethasone treatment with normalization of leukocytosis within 24 hours. Biomarkers (troponin, lactate dehydrogenase/LDH, and CK-MB) normalized within 48 hours, and progressive resolution of ECG changes by 96 hours. The patient had been started on oral cefuroxime at the onset of the sore throat prior to this, which showed little improvement [17]. In our case, NSAIDs were initiated on the day of presentation; the aspirin dose increased from 81 to 1300 mg on day two and continued until the day of discharge. As shown in Table 1, there was little improvement in temperature, leukocytosis, inflammatory markers (ESR and CRP), or troponin. The lack of improvement with six days of NSAIDs and Abx, followed by complete resolution within 24 h of steroids, suggests that autoimmune factor exceeds direct toxin effect.

In 2005, the World Health Organization (WHO) published calculations of over 600,000 million new cases of GAS pharyngitis reported annually. For serious GAS disease, there was a global burden of 18.1 million per year, 1.78 million new cases per year, and 500,000 associated deaths per year, highlighting the far-reaching repercussions of untreated GAS pharyngitis. The disease burden attributed to serious GAS disease is nearly exclusively due to RHD and RHD-related endocarditis and stroke. Although GAS pharyngitis and associated sequela continue to occur in developed countries, the most affected groups are those from low-income, impoverished, or developing countries where there is limited access to healthcare, resulting in an underestimation of the global disease burden due to poor data availability and underreporting [6]. As part of the 2015 Global Burden of Disease Study, there was an estimated 33.4 million cases of RHD and 10.5 million disability-adjusted life-years due to RHD in 2015 globally [33]. It should be noted that these most recent figures may also be underestimated as subclinical RHD were excluded from analysis and estimated three to 10 subclinical cases for every clinical case of RHD [34]. A study carried out by WHO’s Department of Reproductive Health and the Clinton Health Access Initiative (CHAI) estimated that a single dose of long-acting benzathine penicillin G (PBG) costs US$0.11 for 1.2 million IU single dose or US$0.20 for 2.4 million IU dose in low- and middle-income countries [34,35].

## Conclusions

There is a high global burden for this disease with wide-ranging impacts from the number of patients affected to the financial toll it accumulates. Though ARF typically presents two to three weeks following pharyngitis, we demonstrated here a case of acute fulminant carditis presenting with active GAS infection, multiple signs and symptoms of ARF, but unable to meet the Jones criteria. This case illustrates a few key concepts. There is a need for further understanding and use of modified Jones criteria by clinicians, if not further modification of diagnostic criteria to recognize this entity. It may also suggest a mechanism of disease similar to that of ARF carditis and therefore may similarly benefit from early antibiotic and glucocorticoid treatment; however, it may be refractory to NSAID treatment. And lastly, it presents an opportunity to enact a protocol using newer clinical tools, i.e., GAS PCR and troponin, to identify appropriate targets for cost-effective antibiotic treatment to decrease the global disease burden of RHD.

## Figures and Tables

**Figure 6:**
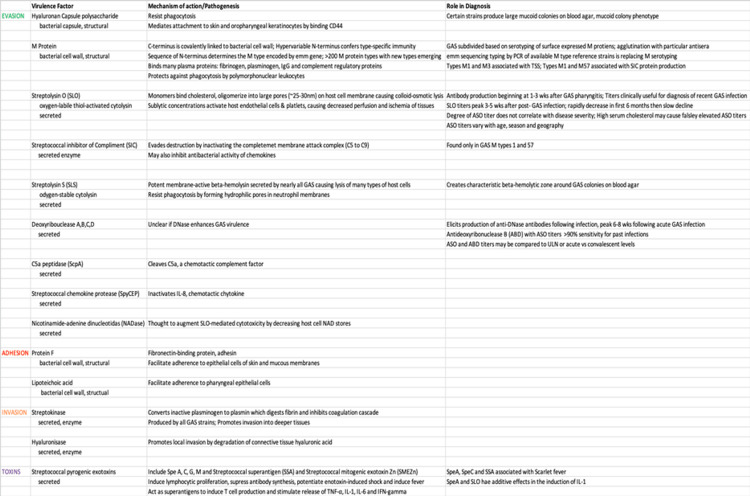
Group A beta-hemolytic streptococcus virulence factors detailed

## Appendices

Appendix A 
